# The Influence of the Various Deposits upon the Teeth and Adjacent Parts

**Published:** 1889-10

**Authors:** Alice L. Sherman


### The Influence of the Various Deposits upon the Teeth and Adjacent Parts.

BY ALICE L. SHERMAN.

These deposits consist of accumulations of food and mucus, serumnal calculus, salivary calculus in its different forms, the green stain, and nicotine. Mingled food and mucus is the deposit occurring most frequently on the teeth; indeed, it is seldom entirely absent. As this becomes lodged near the margin of the gums, on roughened enamel, or between the teeth, and is subjected to the warmth and moisture of the mouth, it undergoes fermentation, forming acids immediately concerned in the production of caries.

If the food is nitrogenous, nitric acid, the chief agent in white decay, is liberated. Sulphurous acid is produced by the decomposition of the albumen of the mucus and of albuminous foods, and this, uniting with the tooth material, causes black decay. Hydrochloric acid, formed by the decomposition of the soluble chlorides of the saliva and mucus, dissolves the inorganic part of the tooth and leaves the organic portion, which appears as brown decay.

These acids, formed in close contact with tooth structures and possessed of remarkable activity on account of their nascent condition, are especially injurious. They predispose to, if they do not actually cause, caries. Further, an accumulation of food may become a nucleus for the formation of salivary calculus, or may itself irritate the gums, inducing a tumid condition.

Serumnal calculus is easily distinguished from salivary calculus; the name itself furnishes the key to the distinction. The first is always preceded by local disease. It is the result of an inflammation in which there has been an infiltration of the tissues about a tooth and extravasation of the serum into the space between the gum and the neck of the tooth, or between the peridental membrane and the cementum. It is from this extravasated serum that the calculus is precipitated. "It is nowhere limited in its beginnings or in its greatest accumulations to the neighborhood of the ducts of the salivary glands."

Serumnal calculus occurs under the margin of the gums in the form of a hard, brown crust. It is also deposited in nodules, adhering very firmly to the necks of the teeth, and sometimes it extends to the apex of the root. This calculus is first produced by local irritation of any kind, but, "a slight deposit, once taken place, becomes an irritant which will, itself, perpetuate the disease."

The adjacent gum and peridental membrane is thus kept in a state of chronic inflammation and the deposit increases. After a time this causes ulceration and destruction of the peridental membrane, exposure of the neck of the tooth, destruction of the alveolar wall and recession of the gum; though in serumnal calculus, the latter suffers less than the peridental membrane and alveolus. As the disease progresses all the symptoms become aggravated; there is a continual flow of pus, the teeth become loosened, and are held only by the thickening of the remaining portion of the peridental membrane.

Salivary calculus varies greatly in color, density, and amount. The largest deposits exist on the buccal surfaces of superior molars and on the lingual surfaces of inferior incisors.

It is generally believed that salivary calculus itself *is not directly injurious* to the teeth. In some cases, indeed, it forms a temporary stopping which arrests caries. Salivary calculus, however, irritates the tissues. There is a tendency to active inflammation of the surrounding parts, producing the same final results as serumnal calculus, *i. e.*, recession of gums, destruction of peridental membrane and alveolus, flow of pus, loosening and loss of the teeth themselves. The destructive effect depends not so much on the amount of the deposit as its distribution.

The lingual surface of the inferior incisors may carry a large load of deposit without producing much irritation, but if the proximal or labial surface is covered, there will be great absorption of gums and destruction of peridental membrane—often exposing the root of the tooth for its entire length.

Sometimes calculus forms rapidly into a coherent mass, completely hiding the anterior teeth. Salter describes a specimen in which "eight anterior lower teeth were encased in a prodigious mass, leading to ulceration of the gums and absorption of the alveolar process, so that these teeth and calculus ultimately fell from the mouth adherent together." By some calculus is regarded as the commonest cause of the loss of the lower teeth in the front of the mouth. Dr. G. V. Black, in an able paper on this subject in the *American System of Dentistry*, says: "Calcic inflammation is really one of the most grave diseases of the teeth. Within my observation it is causing the loss of more teeth than caries. The susceptibility of the gums and other tissues to morbid impressions is influenced to a considerable extent by the constitutional health. Tartar, that in a healthy person would only give rise to slightly increased vascular action in the margin of the gums, in a scorbutic subject would cause them to assume a dark, purple appearance, to become swollen and flabby, recede from the necks of the teeth, bleed, and exhale a fetid odor. Occasionally in persons of the last temperaments, salivary calculus, however small in quantity, is prejudicial."

Bearing these predisposing conditions in mind we can sum up the influence of calculus by giving the whole list of disorders that it induces, any one, or all of which, may result in a given case. They are: a bad breath, inflamed and sensitive gums with hemorrhage, pain, sensitive dentine, absorption of gums and peridental membrane, necrosis and exfoliation of alveolus and maxillæ, loosening and loss of teeth, catarrh, cough, diseases of the antrum, earache, headache, and melancholy. The next deposit which we have to consider is the green stain, found upon the anterior teeth of children near the lunated margin of the gums. This is probably a deposit from the acid-mucus of the mouth. It leaves the enamel roughened, sometimes pitted, and frequently eroded into the very substance of the dentine. The stain, itself, is supposed to be a harmless, granular mass, deposited from an injurious acid secretion.

Finally, there is a dark brown or black deposit, which occurs in the mouths of habitual smokers. It drapes in mourning the lingual surfaces of the upper molars, causes retraction of the gums, and produces a softened condition of the material of the teeth. The latter favors mechanical abrasion.

